# Microtubule-Stabilizing Drugs from Marine Sponges: Focus on Peloruside A and Zampanolide

**DOI:** 10.3390/md8041059

**Published:** 2010-03-31

**Authors:** John H. Miller, A. Jonathan Singh, Peter T. Northcote

**Affiliations:** 1 School of Biological Sciences and Centre for Biodiscovery, Victoria University of Wellington, PO Box 600, Wellington, New Zealand; 2 School of Chemical and Physical Sciences and Centre for Biodiscovery, Victoria University of Wellington, PO Box 600, Wellington, New Zealand; E-Mails: jonathan.singh@vuw.ac.nz (A.J.S.); peter.northcote@vuw.ac.nz (P.T.N.)

**Keywords:** mycalamide, pateamine, peloruside, zampanolide, microtubule stabilization

## Abstract

Marine sponges are an excellent source of bioactive secondary metabolites with potential therapeutic value in the treatment of diseases. One group of compounds of particular interest is the microtubule-stabilizing agents, the most well-known compound of this group being paclitaxel (Taxol^®^), an anti-cancer compound isolated from the bark and leaves of the Pacific yew tree. This review focuses on two of the more recent additions to this important class of drugs, peloruside A and zampanolide, both isolated from marine sponges. Peloruside A was isolated from *Mycale hentscheli* collected in New Zealand coastal waters, and it already shows promising anti-cancer activity. Two other potent bioactive compounds with different modes of action but isolated from the same sponge, mycalamide A and pateamine, will also be discussed. The fourth compound, zampanolide, most recently isolated from the Tongan sponge *Cacospongia mycofijiensi*s, has only recently been added to the microtubule-stabilizing group of compounds, and further work is in progress to determine its activity profile relative to peloruside A and other drugs of this class.

## 1. Introduction

### 1.1. Drugs from marine sources

Despite significant medical advances, cancer continues to be a major cause of disease-related death in most countries. New therapeutic options to treat this disease are a high priority for many pharmaceutical companies and independent research organisations. Current drug discovery involves screening for potential lead compounds with known targets, few side effects, and activity against one or more diseases. There is then potential to remodel the lead compound to improve activity and expand its therapeutic window. *De novo* design and synthesis of bioactive molecules, even with sophisticated computer modelling, is unlikely to replace the millions of years of evolution and natural selection available to living organisms, and thus, natural products, particularly those from marine sources, are still the most promising approach to new drug discovery [1–7]. The diversity of marine life can be attributed to the fact that oceans cover most of the earth’s surface, and marine organisms pre-date terrestrial organisms by hundreds of millions of years, allowing for greater evolutionary variation [8,9]. The success of marine-derived drugs in the clinical treatment of cancer has been particularly encouraging [10–12]. Of the many different types of marine organisms used as a source for drug discovery, sponges have proven to be one of the most successful groups [13], followed by others such as marine bacteria, coelenterates, tunicates, and bryozoans. Marine-derived drugs are generally more potent than plant-derived compounds because of the dilution effect of the ocean environment. Determining the actual source of the bioactive secondary metabolites is an interesting problem; however, the chemical structures of the metabolites provide clues to the metabolic pathways involved in their biosynthesis. Marine sponges harbour microorganisms on their surfaces, in their canal systems, and in their intercellular spaces, and these may contribute up to 40% of the total cellular content of a sponge. Most of these microorganisms are endosymbionts and are probably the major source of the compounds isolated from marine sponges [14–18]. Some indirect evidence for this is the fact that a species of sponge that produces a compound often shows considerable variability between individuals and locations, ranging from some that produce no compound to others that produce large amounts of the compound [19,20].

### 1.2. Cytoskeletal targets

The actual targets of a number of the compounds isolated from marine organisms, including some of those in clinical use, are unidentified, and further work on these compounds is required to understand their mode of action. A common target of bioactive compounds in eukaryotes, however, is the cytoskeleton, largely because it has a major role in many essential cell processes including cell division, cell movement, and cell secretion. The cytoskeleton consists primarily of microtubules, microfilaments, and intermediate filaments. Microtubules and microfilaments are the most vulnerable, and many bioactive compounds target these two structures [21–26]. Microfilaments consist of G-actin monomer proteins polymerized into long, thin F-actin filaments. Examples of marine-derived drugs that target the actin cytoskeleton and cause depolymerization of the filaments are latrunculin A, halichondramide, mycalolide, and the swinholides. Marine drugs that induce or support actin polymerization include phalloidin, jasplakinolide, and dolastatin. The other main cytoskeletal target, the microtubule, consists of polymers of α- and β-tubulin dimers, and there are drugs that either promote tubulin polymerization (microtubule-stabilizing agents) (MSAs) or promote depolymerization (microtubule-destabilizing agents) (MDAs). Paclitaxel was the first MSA discovered and translated into the clinic as an anti-cancer drug, being approved by the U.S. FDA for treatment of ovarian cancer in 1992 [27]. More recent work with paclitaxel involves alterations in its formulation and delivery in attempts to maximise its effectiveness and decrease its toxicity [28]. There are a number of excellent reviews on drugs that target the microtubules and microfilaments in eukaryote cells [21–26,29].

### 1.3. Microtubule-targeting drugs

**Figure 1:**
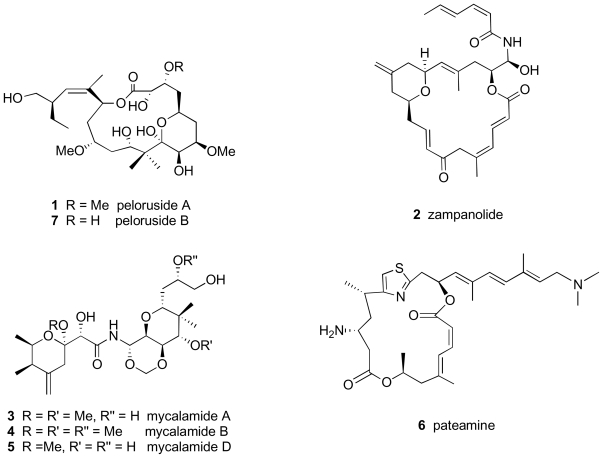
Structures of the compounds.

The number of marine drugs that have been identified as microtubule targeting agents (MTAs) has increased considerably over the years. The first drugs identified as MTAs such as colchicine and the *Vinca* alkaloids, both derived from plants, were found to depolymerize microtubules [23]. Other MTAs have been isolated from non-marine organisms, such as paclitaxel, isolated from the Pacific yew tree *Taxus brevifolia* [30,31], and its semi-synthetic derivative docetaxel, the steroid MDA taccalonolide isolated from the plant *Tacca chantrieri* [32], and the MSAs epothilone A and B, isolated from myxobacteria [33]. Other MSAs are derived from marine sources but not from sponges, including eleutherobin [34,35] and sarcodictyin [36,37], both from soft corals. Marine sponges, however, remain the most prolific source of MTAs [24–26] and include the MDAs jaspolide, dolastatin, halichondrin, spongistatin, and milnamide and the MSAs hemiasterlin, dictyostatin, discodermolide, laulimalide, peloruside A, and zampanolide. The present review will focus on the last two of the MDAs listed above, peloruside A (**1**) and zampanolide (**2**) (Figure 1). The history of their isolation, purification, and the determination of their mode of action will be discussed. In addition, two other bioactive compounds collected from the same sponge that produces peloruside A but that does not target the cytoskeleton, the mycalamides **3**–**5** and pateamine (**6**), will also be discussed in conjunction with peloruside A.

## 2. Discussion

### 2.1. Peloruside A and its cell-mates, mycalamide A and pateamine

**Figure 2:**
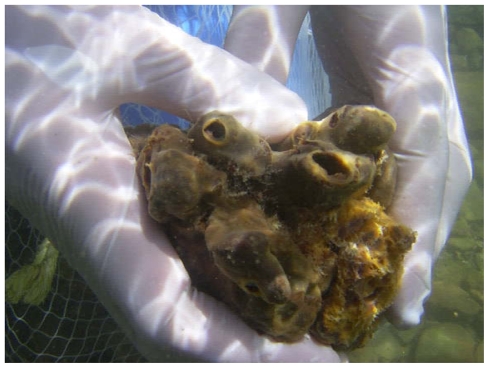
The marine sponge *Mycale hentscheli* collected from Pelorus Sound, New Zealand.

The history of lead compound development from marine natural sources is a fascinating aspect of drug discovery. Dr Peter Northcote, working with Dr John Blunt and Dr Murray Munro at the University of Canterbury in Christchurch, and then later in his own laboratory at Victoria University of Wellington, was instrumental in the identification of two of three bioactive secondary metabolites isolated from a single species of marine sponge *Mycale hentscheli* from New Zealand coastal waters (Figure 2). The sponge produces three structurally dissimilar classes of secondary metabolites with potent bioactivity in the low nanomolar range, the mycalamides **3**–**5**, pateamine (**6**), and the pelorusides A (**1**) and B (**7**) [38–40] (Figure 1).

### 2.2. Mycalamides

Mycalamide A (**3**) was originally isolated from *Mycale sp.* marine sponges collected in Otago Harbour in New Zealand by Perry *et al.* in 1988 using a bioactivity-directed isolation protocol [41]. Mycalamide analogues B (**4**) and D (**5**) have also been identified in *Mycale* marine sponges [42,43]. A related compound, mycalamide C, was reported from a *Stylinos* sp. sponge [44]. The mycalamides have a high degree of structural similarity to pederin, a toxin isolated from the terrestrial blister beetle *Paederus fiscipes* [45–47], as well as the onnamides and theopederins, isolated from *Theonella and Discodermia* marine sponges [48–54]. The structures of mycalamides A, B, and D, **3**–**5**, are shown in Figure 1. Mycalamide A was found to be a potent inhibitor of cell proliferation (IC_50_ 1–5 nM) [38,42,55] and, like onnamide A, inhibits *in vitro* protein translation at concentrations close to those required to inhibit cell proliferation [55]. Mycalamide B (**4**), with three oxymethyl groups instead of two, is a more potent inhibitor of both cell proliferation and *in vitro* translation than mycalamide A, suggesting that an additional oxymethyl group increases the affinity of binding to the cellular target, rather than promotes uptake by the cell. Another mycalamide congener isolated from the same sponge species, mycalamide D (**5**), with one oxymethyl group, is less potent than mycalamides A or B [43], and this decrease in activity may result from the increased polarity of the analog and therefore decreased entry into the cell. The ability of pederin, with structural similarities to the mycalamides, to inhibit protein synthesis by binding irreversibly to ribosomes was originally described by Carrasco *et al.* in 1976 [56]. Closer examination of the structure of pederin revealed a region in the molecule similar to a peptide bond, and this may explain the protein synthesis inhibitor properties of pederin [57]. Both pederin and mycalamide bind to the large ribosomal subunit of eukaryotic cells [58].

The total synthesis of mycalamide A has been described [59–63], as well as syntheses of a number of biologically active analogs and semi-synthetic derivatives [57,64–68]. The synthetic analogue 18-*O*-methyl mycalamide B is slightly more potent than mycalamide A and inhibits [^125^I]-uridine and [^3^H]-leucine incorporation into RNA and protein in cultured cells [57]. In animal studies, mycalamides A and B increased the life span of mice bearing ascitic P388 lymphoma and other solid tumours, although significant side effects were observed [55]. Since this initial study, further *in vivo* studies with the mycalamides have not been reported in the literature.

### 2.3. Pateamine

Pateamine (**6**) (Figure 1) was reported from sponges collected in Fiordland, New Zealand by Northcote *et al.* in 1991 and found to be highly toxic against P388 leukemic cells (IC_50_ = 0.15 ng/mL), as well as exhibiting anti-fungal properties [69]. Romo *et al.* described the first synthesis of pateamine in 1998 and also noted its immunosuppressive properties [70]. Pateamine was proposed to be a protein synthesis inhibitor, based on the early work with the compound. In one study, pateamine was chemically modified by the addition of dexamethasone to the primary C-3 amino group to allow yeast-three hybrid screens to be performed to identify the cellular receptor of the drug [70,71]. To our knowledge, however, no further work using this approach has been published. The cellular target of pateamine was subsequently identified by affinity chromatography to be the protein initiation factor eIF4A [72–74]. By stimulating eIF4A, pateamine inhibits protein synthesis. Interestingly, it was also shown to inhibit nonsense-mediated mRNA decay [75]. This second effect was also *via* eIF4A, but the mRNA decay was independent of the translation inhibition effects of the drug. Thus, pateamine is a novel small molecule inhibitor of protein synthesis that works by binding to and stimulating a eukaryote translation initiation factor. As such, it is an important molecular tool for studying the biochemical events of protein synthesis initiation and mRNA turnover.

### 2.4. Peloruside A

#### 2.4.1. Discovery and mode of action

**Figure 3:**
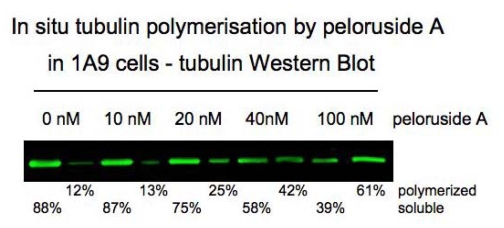
Peloruside A-induced tubulin polymerization in 1A9 ovarian carcinoma cells. Cells were treated with peloruside A and a supernatant and pellet fraction electrophoresed. The gel was immunoblotted for α-tubulin. Image supplied by Arun Kanakkanthara.

The third secondary metabolite from *Mycale hentscheli* that showed potent bioactivity was isolated in 1999 from sponges collected from Pelorus Sound on the north coast of the South Island of New Zealand [76]. This novel compound was named peloruside A (**1**) after the location where the sponges were found (Figure 1). Dr Northcote and his PhD student at the time, Lyndon West, identified peloruside A using a unique fractionation procedure that enriched for bioactive compounds on the basis of their intermediate solubility in aqueous methanol, being neither too lipophilic nor too hydrophilic. Peloruside A was initially found to be ten-fold less potent an inhibitor of proliferation of P388 murine leukemic cells (IC_50_ = 18 nM) [76] than the two other natural products isolated from the same sponge, mycalamide A and pateamine. Although all three metabolites are cytotoxic at nanomolar concentrations, the mechanism of action of peloruside A differed considerably from the other two which were known protein synthesis inhibitors. Shortly after the structure of peloruside A was determined and published [76], Dr Northcote and Dr John Miller, a cell biologist at the same university, began collaborating together to identify new natural products and determine their modes of action. Although initial studies on the mechanism of action of peloruside A followed up on a false lead that was based on structural similarity of the active sites on peloruside A to those of bryostatin-1 [39], Kylie Hood, then a PhD student of Dr Miller’s, discovered that peloruside A had potent anti-mitotic activity, blocking cells in G_2_/M of the cell cycle [39,40]. Further investigations determined that peloruside A stabilized the polymerized form of tubulin (Figure 3) and induced microtubule bundling in interphase cells and multiple asters in mitotic cells [77] in a similar manner to paclitaxel [31], an important anti-cancer drug used for the treatment of solid tumours of the breast, ovary, lung, head and neck [27,78]. The history of the discovery and development of paclitaxel as an anti-cancer agent has been summarised by Prof. Susan Band Horwitz in a special article to commemorate her impact in the field, published in the *Journal of Natural Products* [79].

In quantifying the activity of different MSA’s, the critical concentration (C_crit_) is a useful comparative parameter. C_crit_ is the minimum concentration of tubulin that is required for polymerization to occur. GDP-tubulin in the absence of an MSA does not polymerize at all *in vitro* (C_crit_ > 200 μM) [80]. The C_crit_ for tubulin in the presence of peloruside A in a MAP-free *in vitro* system is higher than for tubulin in the presence of paclitaxel (11 μM versus 4 μM, respectively), indicating that it is a less potent MSA than paclitaxel [81]. Nevertheless peloruside A is capable of polymerizing microtubules in the absence of MAPs. Both the presence of GTP and MAPs can influence the C_crit_ for tubulin. For example, in the presence of paclitaxel, the C_crit_ for tubulin with MAPs and GTP present, MAPs only, GTP only, and in the absence of both MAPs and GTP is 0.2, 2, 4, and 22 μM, respectively [82]. The C_crit_ for tubulin after addition of peloruside A was only tested in the absence of MAPs and in the presence of GTP [81].

Recent studies in Dr Miller’s laboratory have also demonstrated that, like other MSA’s [83–86], peloruside A inhibits microtubule dynamics at concentrations near those needed to block progression through the cell cycle, but much lower than those needed to cause tubulin assembly *in vitro* [87]. Although not tested in mitotic cells, peloruside A significantly reduced the growth and shortening of microtubules in interphase cells, reducing the overall dynamicity of the microtubules.

#### 2.4.2. Peloruside A effects on cell proteins

A proteomics study on human HL-60 promyelocytic leukemic cells used two-dimensional electrophoresis and differential in-gel electrophoresis (DIGE) to examine the protein changes in the cell following treatment with peloruside A [88]. An analysis of the affected proteins showed 17 proteins changed by 2-fold or more, and the changes were consistent with the known action of peloruside A on microtubules, as well as effects on proteins involved in apoptosis and stress. Others have also shown that blocking cells in mitotsis with anti-mitotic drugs of different types leads to activation of apoptosis, often through interactions with the transcription factor c-Myc and the tumor suppressor protein p53 [89]. A connection with c-Myc protein action was indicated in the proteomic study by Wilmes *et al.* [88], with c-Myc being decreased in abundance by peloruside A. Many of the off-target proteome changes either result from downstream effects on the primary target or effects on secondary targets, both of which could contribute to side effects of the drug *in vivo*.

#### 2.4.3. Peloruside A binding site on tubulin

An interesting study by Pryor *et al.* in 2002, working with laulimalide, a relatively new MSA, showed that more than one binding site existed for the stabilizing compounds [90]. Laulimalide bound to a different site to that of the classic MSA’s, paclitaxel, epothilone, and discodermolide, which all bind to the well-described taxoid site on β-tubulin and can compete for each other’s binding [91,92]. Peloruside A was also shown to have a different binding site on the tubulin dimer to paclitaxel but was seen to bind to the same or an overlapping site with laulimalide [81]. This information on the peloruside A binding site was obtained through a collaboration beginning in 2004 beween Drs Miller and Northcote with two specialists in microtubule protein structure and function, Dr Fernando Díaz and Dr José Andreu of the Centro de Investigaciones Biológicas in Madrid. In a Flutax-2 (fluorescently-labeled paclitaxel derivative) displacement test using MALDI-TOF mass spectrometry, concentrations of up to 2,000-fold excess of peloruside A over Flutax-2 failed to displace Flutax-2 from the taxoid binding site; whereas, a 100-fold excess of paclitaxel could compete for the Flutax-2 binding completely. Laulimalide was also tested, and a 10-fold excess was found to compete for binding with peloruside A but not for Flutax-2 binding. Thus, peloruside A and laulimalide were proposed to have the same or an overlapping binding site [81]. Indirect evidence from this same study of a distinct binding site for peloruside A was its lack of sensitivity to resistance conferred by mutations to the taxoid binding site. Further work in collaboration with Dr Ernest Hamel at the National Cancer Institute (NCI/NIH) in Frederick, MD demonstrated that peloruside A could synergize with the taxoid site drugs in causing tubulin polymerization *in vitro* [93] and in cells [94], but peloruside A was unable to synergize with laulimalide, at least *in vitro*, thus, supporting the novel peloruside/laulimalide binding site on tubulin. The exact binding site on the tubulin dimer has still not been unequivocably identified at this stage, although a recent study using hydrogen-deuterium exchange mass spectrometry by Dr David Schriemer’s group in Calgary, Canada has proposed a peloruside A binding site on β-tubulin that is distinct from the taxane site [95]. Two previous studies that used computer docking also modeled the peloruside/laulimalide binding site, proposing it to be on α-tubulin rather than β-tubulin [96,97]. The question of an α- or β-tubulin binding site still needs to be resolved. It is possible of course that both binding sites exist, and further work is necessary in this area to settle this question. Dr Miller and Northcote are currently working with Dr Paraskevi Giannakakou at Weill Medical College of Cornell in New York City and Prof Jim Snyder at Emory University in Atlanta, using computer modeling and peloruside/laulimalide resistant cells to address this question.

At Victoria University of Wellington, experiments are also in progress with Dr David Bellows, a yeast geneticist, on the peloruside A binding site on yeast tubulin, taking advantage of the accessibility of the yeast genome and the ability to generate resistant mutants by site-directed mutagenesis [98]. The aim of this research is to map the peloruside A binding site by altering specific amino acids in yeast tubulin. Similar types of experiments on the taxoid binding site have been carried out by Prof Richard Himes’ group at the University of Kansas [99,100]. It was found that paclitaxel was unable to bind to yeast tubulin without humanization of the taxoid site sequence by converting five amino acids to those found in the human tubulin gene. Chemical genetics offers a new, exciting opportunity for identification of the targets of novel marine drugs, since chemical genetic screens are relatively easy to carry out in yeast, using genome-wide synthetic lethal arrays [101,102]. There is significant homology between yeast and mammalian cells, and a target identified in yeast has a good chance of having a homolog in mammalian systems. Similar types of screens in mammalian cells are possible using siRNA gene silencing techniques [103,104]. Whitehurst *et al.* looked at the effects of paclitaxel on growth of a human non-small-cell lung cancer cell line and interestingly identified α-tubulin as one of its hits, although its binding site is on β-tubulin, and also a number of proteasome genes. Network analysis from genome-wide screens has introduced the interesting possibility that drugs may target networks rather than single proteins [105,106], and this concept to some extent may help explain the multiple effects seen when a single drug is added to cells.

Paclitaxel is known to bind to the β-subunit of the microtubule at the taxoid binding site situated in a pocket lined by several hydrophobic residues [91,92,107]. The pocket is at the boundary between the nucleotide-binding domain and the middle domain of β-tubulin, and is in contact with the M-loop of tubulin. Paclitaxel greatly inhibits the peeling apart of protofilaments, suggesting that it stabilizes the microtubule through strengthening lateral interactions between the strands. Another possibility is that paclitaxel reduces the ability of protofilaments to curl, a process that occurs normally. A third possibility is that paclitaxel stabilizes the conformation of tubulin, stopping the movement of the nucleotide-binding domain against the central tubulin domain. Recent hydrogen-deuterium exchange mass spectrometry studies with discodermolide and chicken tubulin by Prof. Susan Horwitz’s group at Albert Einstein College of Medicine in New York have predicted a different conformation and stabilization mechanism to paclitaxel, although both bind in the same region [107]. Unlike paclitaxel, discodermolde does not bind to the M-loop but is turned toward the N-terminal H1-S2 loop on the opposite side of the taxane binding pocket which is proposed to be involved in the lateral contact between protofilaments. Although both drugs stabilize the interdimer and interprotofilament interactions, the mechansim of the interaction thus differs, with discodermolide having a greater effect on the interdimer interactions [107]. The conformational changes resulting from the different interdimer stabilizations share the potential to counteract the curvature of protofilaments, thus stabilizing the polymer. Since chicken tubulin has some amino acid differences from human or bovine tubulin in or close to the region of the taxoid binding site [108], there may be differences in the detailed interactions of paclitaxel and discodermolide with amino acids to those occurring in binding to mammalian tubulin.

In 2008, Huzil *et al.* [95] suggested that the mechanism of microtubule stabilization by peloruside A differs from stabilization by these taxoid-site drugs. This is not surprising, given its distinct binding site. Taxoid-site drugs interact with the βH7 region (bottom of the taxoid-binding cleft) that lies next to the T5 loop at the exchangeable nucleotide binding region; whereas, peloruside A interacts with the H9-S8 loop at the proposed binding site of peloruside A [95]. This binding site also lies next to the T5 loop. A conformational change in the T5 loop is believed to promote improved interactions across the interdimer interface, causing stabilization.

#### 2.4.4. Peloruside congeners and analogues

In addition to work with natural congeners, including peloruside B (**7**) (Figure 1) [109], which has similar bioactivity to peloruside A, studies are in progress on semi-synthetic modifications to the structure of peloruside A. Opening of the pyranose ring of peloruside A by sodium borohydride reduction increased the IC_50_ for cell growth inhibition by 26-fold in a murine leukocyte cell line (32D) [39]. Attempts to modify peloruside A and attach a fluorescent or radioactive label have not been overly successful due to the complexity of the molecule. Recently, however, a tritiated derivative of peloruside A has been prepared. Dr Ernest Hamel at NCI/NIH has used this ^3^H-peloruside A to examine competitive binding to microtubules (personal communication, 2010). Some modifications to the C-24 side chain have been carried out, and the activity of the side chain analog is currently under investigation in the laboratories of Drs Miller and Northcote, Dr Díaz, and Prof Ian Paterson in the Chemistry Department at the University of Cambridge.

#### 2.4.5. Anti-disease potential of peloruside A

Peloruside A is currently under development as an anti-cancer drug through an agreement between Victoria University of Wellington and REATA Pharmaceuticals Inc., Dallas, Texas. Victoria University holds the patent on peloruside A [110]. Peloruside A has some promising advantages over paclitaxel, being more soluble and therefore not requiring the use of Cremophore EL, a polyoxyethylated castor oil, to deliver the drug to the body. This should correlate with fewer vehicle-associated side effects than paclitaxel [78]. Peloruside A is also more likely to be effective against cells that acquire the multiple-drug resistance (MDR) phenotype, since peloruside A remains active in cells with high P-glycoprotein drug efflux pump expression [81]. The resistance ratio of peloruside A in cells with an MDR phenotype to wild type cells was 21 in this study, compared with 1417 for paclitaxel [81]. Tests of peloruside A in animals have been very promising, with peloruside A showing no overt toxicity in mice and being more efficacious in inhibiting tumor growth than paclitaxel and docetaxel [111].

Recent work with a mouse model of multiple sclerosis (experimental autoimmune encephalomyelitis) by Dr Anne La Flamme at Victoria University of Wellington has demonstrated that peloruside A is as effective as paclitaxel at delaying the onset of the disease but, unlike paclitaxel, shows no toxicity [112]. Work has also begun at Victoria University of Wellington on the effect of peloruside A on microtubule-associated proteins and the potential value of the compound for the treatment of neurodegeneratory diseases like Alzheimer’s disease. Studies with MSA’s have already shown promise in this area since paclitaxel treatment can rescue motor function in animals with loss of tau protein activity [113].

At the present time, preclinical studies and the advancement of peloruside A into Phase I clinical trials for cancer therapy are being held back due to the short supply of natural and synthetic peloruside A. Attempts to aquaculture *Mycale hentscheli* in New Zealand coastal waters were initially very successful [19,20], but later were discontinued as a result of devastation of the sponge cultures by invading nudibranchs. An interesting finding from the aquaculture studies was that individual sponge samples that have good levels of peloruside A maintained those high levels in culture; whereas, samples that originally lacked peloruside A never acquired the ability to produce peloruside A, even when cultured in close proximity to peloruside A-producing sponges.

The De Brabander group at the University of Texas Southwestern was the first to carry out a total synthesis of peloruside A [114]. This first synthesis produced the inactive enantiomer of peloruside A, but the group subsequently re-synthesized the correct, bioactive enantiomer. Since then, three other laboratories have synthesized the compound in milligram amounts [115–117]. The synthetic strategies for peloruside A have been reviewed by Williams *et al.* [118]. A congener of peloruside A, peloruside B (**7**), has recently been synthesized by the Ghosh laboratory at Purdue University [109].

### 2.5. Zampanolide

#### 2.5.1. Discovery and synthesis

**Figure 4:**
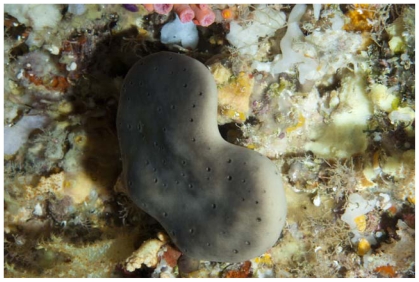
*Cacospongia mycofijiensis*, collected from Vava’u, Tonga. Photograph courtesy of Karen Stone, Dive Vava’u.

After having worked on peloruside A for over 10 years, in 2009 Dr Northcote along with his PhD student Jonathan Singh found another sponge natural product, zampanolide (**2**) (Figure 1) in a collection of sponges taken from an underwater cave off the Tongan coast. The sponge, *Cacospongia mycofijiensis* (Figure 4), generated a number of different bioactive compounds, all of which had been described previously, including laulimalide and isolaulimalide (MSA’s), latrunculin A (an actin-destabilizing agent), dendrolasin (also isolated from insects and having pheromone activity), mycothiazole (function unknown), and zampanolide (function unknown at the time). Three Japanese chemists from Okinawa and Kyoto had just published a paper describing the synthesis and isolation of zampanolide from the sponge *Fasciospongia rimosa* [119]. Tanaka and Higa, however, were the first to describe the structure of zampanolide thirteen years earlier [120]. This 20-membered macrolide has potent, low nanomolar inhibitory activity on cell growth. Amos Smith, III at the University of Pennsylvania also published a total synthesis of the enantiomer of zampanolide [121].

#### 2.5.2. Mode of action

**Figure 5:**
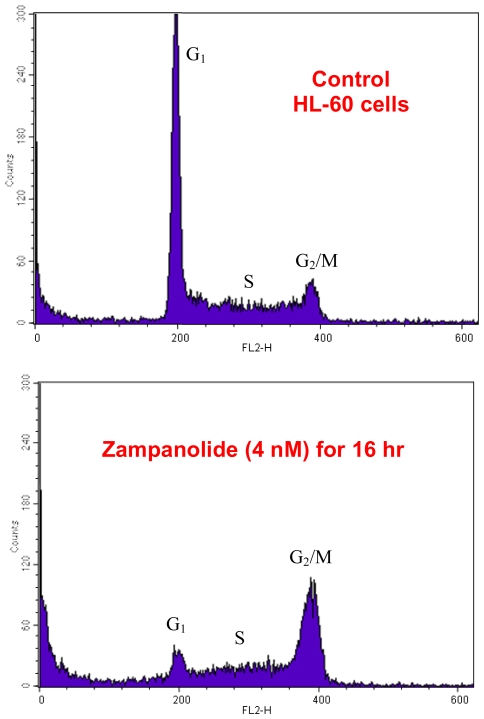
Effect of zampanolide on cell cycle progression using flow cytometry. HL-60 promyelocytic leukemic cells were treated with 4 nM zampanolide for 16 hr, stained with propidium iodide, and analyzed by flow cytometry. Graphs courtesy of Jessica Field.

Since zampanolide was a very potent bioactive compound with an IC_50_ for growth inhibition in the low nanomolar range, Drs Miller and Northcote decided to have a look at its mechanism of action. As luck would have it, a simple flow cytometry experiment carried out at Victoria University of Wellington by Dr Miller’s graduate student Jessica Field (Figure 5) showed a clear block in the G_2_/M phase of the cell cycle, suggesting an anti-mitotic action and a possible cytoskeletal target, either microtubules or microfilaments. It was also possible, of course, that the new compound interacted with a regulator of cell division, such as a cdc kinase or other mitotic regulatory protein; thus, there was no guarantee that its mode of action would be easily solved.

After the earlier groundwork with the MSA peloruside A in the Miller and Northcote laboratories, further experiments were carried out to check cytoskeletal activity of zampanolide, and the target was fairly quickly singled out as the microtubule. Confirmation that zampanolide was a new MSA was subsequently demonstrated by testing for intracellular polymerization of tubulin in cultured cells, the induction of microtubule bundling in interphase cells, and the generation of multiple asters and micronuclei during mitotic division. Finally, zampanolide was shown to promote extracellular tubulin assembly with purified bovine tubulin in the absence of added microtubule-associated proteins. The confirmed microtubule-stabilizing mode of action of zampanolide and the isolation and purification details were then published in the *Journal of Medicinal Chemistry* [122] less than six months after the Uenishi *et al*. paper describing the isolation and synthesis of zampanolide appeared on-line in 2009 [119]. It was also reported in the Field *et al*. [122] paper that overexpression of the P-glycoprotein (P-gp) drug efflux pump had no effect on the potency of zampanolide; hence, zampanolide remained effective in cells with an MDR phenotype, an important property for new generation MSA’s destined for clinical development. The other immediate promising characteristic of zampanolide, seen in its structure (Figure 1), was its reduced number of stereogenic centers compared to other MSA’s. Its simplified chemical structure suggested that a scale-up of the reported synthetic protocols [119–121,123–127] would be easier and less costly than for other MSA macrolides, and would improve its chances for entry into clinical trials. Further characterization of zampanolide is now underway at Victoria University of Wellington, the Díaz laboratory in Madrid, the Hamel laboratory at NCI/NIH in Maryland, and the Horwitz laboratory at Albert Einstein in New York to determine whether it binds to the taxoid site on tubulin, the laulimalide/peloruside site, or to a third site not previously described.

## 3. Conclusions

The newest members of the microtubule-stabilizing class of drugs, the pelorusides and zampanolide, show good potential for future development as anti-cancer drugs, and further characterization of their unique activity profiles is underway. The sponges from which these drugs were extracted also produce many other bioactive compounds with differing modes of action, including the mycalamides and pateamine, and thus, marine sponges remain an excellent source of bioactive compounds for potential development as pharmaceuticals for the treatment of cancer and other diseases.
